# A causal inference framework for poststratification: a method for improving external validity in epidemiological studies

**DOI:** 10.1186/s12874-026-02835-y

**Published:** 2026-04-16

**Authors:** Yeon Woo Oh, Dongkyu Lee, Jaelim Cho, Changsoo Kim, Kyoung-Nam Kim

**Affiliations:** 1 Department of Biostatistics and Computing, Yonsei University Graduate School, Seoul, Republic of Korea; 2 Department of Preventive Medicine, Yonsei University College of Medicine, 50 Yonsei-ro, Seodaemun-gu, Seoul, 03722 Republic of Korea; 3 Institute for Environmental Research, Yonsei University College of Medicine, Seoul, Republic of Korea; 4 Institute of Human Complexity and Systems Science, Yonsei University, Incheon, Republic of Korea

**Keywords:** Causal inference, External validity, Epidemiology, Inverse probability weighting, Standardization

## Abstract

**Background:**

Poststratification, a method for improving the representativeness of nonprobability samples, has developed primarily within survey methodology. Meanwhile, g-methods such as inverse probability weighting (IPW) and standardization have been developed in epidemiology for causal inference from observational data. Despite evolving under different terminology in two fields, these methods share similar underlying assumptions and estimation strategies. In this article, we systematically articulate the formal connections between poststratification and g-methods, demonstrating how each field can inform the other.

**Methods:**

We develop a methodological framework demonstrating how poststratification can be understood through established causal inference principles. We formally map the three core assumptions required for valid poststratification onto the identifiability conditions used in causal inference from observational data. We show that the two principal implementation approaches for poststratification parallel inverse probability weighting and standardization. To illustrate the practical application, we apply poststratification to data from the Korean Genome and Epidemiology Study (KoGES) of 10,030 Korean adults aged 40–69 years in 2001, adjusting for age and sex distributions to estimate population-level smoking prevalence.

**Results:**

The formal mapping reveals that poststratification and g-methods share analogous assumptions and estimation strategies. This parallel provides principled guidance for auxiliary variable selection, clarifies when poststratification succeeds or fails, and enables application of established diagnostic tools from causal inference to poststratification problems. In the KoGES example, poststratification adjusted the crude smoking prevalence from 25.7% to 26.5%, accounting for oversampling of older participants.

**Conclusions:**

Understanding poststratification through the lens of causal inference offers a rigorous foundation for making valid population-level inferences from nonprobability samples. This framework facilitates cross-disciplinary learning and enables more principled interpretation of results from convenience samples, cohort studies, and other nonprobability sampling designs. Future work could explore how poststratification methods relate to the broader literature on generalizability and transportability of results from randomized trials.

## Background

Contemporary medical research regards randomized controlled trials (RCTs) as the methodological gold standard, with observational studies striving to approximate randomization through design and analysis. This paradigm has motivated the development of sophisticated methods for causal inference from observational data, including g-methods such as inverse probability weighting (IPW) and standardization. At the same time, most medical studies necessarily rely on nonprobability samples, in which the probability of participants being included in the study is not known a priori, raising concerns about the external validity of study findings.

While large population-based surveys employing probability designs (e.g., the National Health and Nutrition Examination Survey [1]) provide a well-established approach to population-level inference, substantial methodological work has also addressed how to establish external validity from nonprobability samples. One strategy to address the representativeness of nonprobability samples is poststratification [2]. Poststratification is applicable when the distribution of the auxiliary variables is known both in the sample and the target population. The technique reweights sample observations so that auxiliary variable distributions match those of the target population, thereby enabling inference about population-level quantities such as the mean or proportion of the variable of interest [3].

The methodological structure of poststratification closely parallels that of g-methods widely used in epidemiology for causal inference. While these approaches have developed under different names—poststratification in survey sampling and g-methods in epidemiology—they share similar underlying assumptions and estimation strategies [4, 5]. Both endeavors address “nonrandom” problems–whether arising from nonrandom treatment assignment or nonrandom sample inclusion–and both employ weighting or stratification methods to restore balance. Consequently, poststratification can be understood naturally through the causal inference framework familiar to most epidemiologists.

While Mercer et al. explored parallels between survey inference and causal inference in the context of nonprobability sampling, their work focused on survey methodology broadly rather than epidemiological applications and did not examine poststratification specifically [6]. Lesko et al. examined the similarities between improving external validity and causal inference but did not focus on poststratification [4, 5]. The methodological connections between poststratification and causal inference–particularly regarding the underlying assumptions and implementation strategies–have not been systematically articulated in the epidemiological literature.

In this article, we develop a methodological framework demonstrating how poststratification can be rigorously understood through the established principles of causal inference. Our aims are threefold: (1) to formally map the assumptions required for valid poststratification onto the standard identifiability conditions for the ATE under IPW and standardization, (2) to show that the implementation approaches for poststratification correspond to IPW and standardization methods, and (3) to illustrate these theoretical connections using empirical data. By elucidating these parallels, we hope to provide epidemiologists with a more intuitive understanding of poststratification, facilitate principled auxiliary variable selection, and encourage wider adoption of these methods for population-level inference.

## Methods

This methodological framework has been developed through systematic comparison of poststratification and causal inference principles. The theoretical parallel is demonstrated through mathematical derivations mapping the assumptions and implementation methods between the two frameworks.

The framework is illustrated using data from the Korean Genome and Epidemiology Study (KoGES), a population-based cohort study of Korean adults aged 40–69 years (*n* = 10,030, baseline 2001) [7]. Smoking prevalence estimates were calculated using both crude and poststratification-adjusted approaches (individual-level weighting and stratification), with adjustments based on age-sex distributions from the 2001 Korean Population Census.

## Results

### Causal inference framework

We begin by establishing the conceptual foundations of causal inference. For expositional clarity, we assume a binary treatment $\:A\in\:\left\{\mathrm{0,1}\right\}$ and binary outcome $\:Y\in\:\left\{\mathrm{0,1}\right\}$, with confounders $\:X$ represented as categorical variables. $\:{Y}^{a}$ denotes the counterfactual outcome that would be observed if treatment were set to $\:A=a$. The average treatment effect (ATE) is defined as $\:E\left[{Y}^{1}\right]-E\left[{Y}^{0}\right].$.

Identifying the ATE from observational data using standard g-methods such as IPW and standardization requires three core assumptions [8]:

1. Conditional Exchangeability: $\:{Y}^{a}\perp\:A|X$ for all $\:a$. Conditional on covariates $\:X$, treatment assignment is independent of potential outcomes. This condition holds when $\:X$ includes all variables that jointly influence both treatment and outcome.
2. Positivity: $\:P\left(A=a|X=x\right)>0$ for all $\:a$ and $\:x$. Every stratum defined by $\:X$ must contain individuals receiving each treatment level. Without positivity, certain counterfactual quantities cannot be estimated from the observed data, as some treatment-covariate combinations remain empirically unobserved.
3. Consistency: $\:Y=A{Y}^{1}+\left(1-A\right){Y}^{0}$. Consistency ensures that the observed outcome $\:Y$ for a subject receiving treatment $\:A=a$ equals their counterfactual outcome $\:{Y}^{a}$.

To understand these conditions, first consider how RCTs enable causal inference. In an RCT, the ATE is estimated simply as $\:E\left[Y|A=1\right]-E\left[Y\right|A=0]$, the difference in mean outcomes between treatment arms. This estimator is valid because the following equation holds:

1 $$\:E\left[{Y}^{a}\right]=E\left[Y|A=a\right]$$

Why does Eq. (1) hold? Starting from the right-hand side, consistency implies $\:E\left[Y|A=a\right]=E\left[{Y}^{a}|A=a\right]$. Randomization ensures marginal exchangeability; hence, $\:{Y}^{a}\perp\:A$ holds unconditionally. Therefore, $\:E\left[{Y}^{a}|A=a\right]=E\left[{Y}^{a}\right]$. Finally, positivity is guaranteed by design: each treatment arm contains at least some participants, making $\:E\left[Y|A=a\right]\:$estimable from the observed data.

Exchangeability merits particular attention. The equality $\:E\left[{Y}^{a}|A=a\right]=E\left[{Y}^{a}\right]$ states that the average potential outcome in the treated group equals that of the entire study population. This property holds, for instance, when the treated group constitutes a simple random sample (SRS) from the population—which is precisely what randomization achieves. By assigning treatment independently of all participant characteristics, randomization creates treatment groups that are equivalent to random samples from the study population.

In observational studies, confounding precludes direct estimation of the ATE via $\:E\left[Y|A=1\right]-E\left[Y|A=0\right]$. However, when confounders $\:X$ are measured, exchangeability and positivity hold conditional on $\:X$, and consistency is satisfied, the counterfactual mean $\:E\left[{Y}^{a}\right]$ can be estimated through inverse probability weighting (IPW) or standardization [8].

IPW creates a pseudo-population in which treatment assignment is independent of potential outcomes by weighting each observation by the inverse of its treatment probability. Specifically, an individual with covariates $\:X$ receiving treatment $\:A=a$ is weighted by $\:1/P\left(A=a|X\right)$. The weighted average then identifies the counterfactual mean:

2 $$E\left[\frac{Y\cdot\:I\left(A=a\right)}{P\left(A=a|X\right)}\right]=E\left[\frac{{Y}^{a}\cdot\:I\left(A=a\right)}{P\left(A=a|X\right)}\right]$$

3 $$=E\left[E\left[\frac{{Y}^{a}\cdot\:I\left(A=a\right)}{P\left(A=a|X\right)}\Bigg|X\right]\right]$$

4 $$=E\left[\frac{E\left[{Y}^{a}\right|\mathrm{X}]\cdot\:\mathrm{E}[I\left(A=a\right)\left|X\right]}{P\left(A=a|X\right)}\right]$$

5 $$=E\left[E\left[{Y}^{a}|X\right]\right]=E\left[{Y}^{a}\right]$$

where $\:I(A=a)$=1 if $\:A=a$ and 0 otherwise. Conditional exchangeability $\:{Y}^{a}\perp\:A|\:X$ justifies the step from Eq. (3) to (4), where the conditional expectations of $\:{Y}^{a}$ and $\:I(A=a)$ given $\:X$ factorize.

Standardization computes the counterfactual mean $\:E\left[{Y}^{a}\right]$ by first estimating the conditional outcome $\:E\left[Y|A=a,X\right]$ within each stratum of $\:X$ among those receiving treatment $\:A=a$, then averaging these stratum-specific estimates weighted by the population distribution of $\:X$. Formally:

6 $$\sum\limits_{x}E\left[Y|A=a,X=x\right]P\left(X=x\right)=\sum\limits_{x}E\left[{Y}^{a}|A=a,X=x\right]P\left(X=x\right)$$

7 $$=\sum\limits_{x}E\left[{Y}^{a}|X=x\right]P\left(X=x\right)=E\left[{Y}^{a}\right]$$

The step from Eqs. (6) to (7) invokes conditional exchangeability $\:{Y}^{a}\perp\:A\:|\:X$, which permits the removal of conditioning on $\:A\:$within each stratum. This means that while treatment and counterfactual outcomes may be associated marginally, this association vanishes within strata of $\:X$. Within each stratum, comparing outcomes between treated and untreated individuals yields causally valid inferences.

### Poststratification framework

We now describe the mechanics of poststratification. Under SRS, the sample distribution of any variable matches its distribution in the target population in expectation. Conversely, when sampling is nonrandom, certain variables may exhibit distributional discrepancies between the sample and population. Poststratification corrects such discrepancies by reweighting observations to align the sample distribution of auxiliary variables with their known population distribution.

Consider estimating the obesity rate in a target population from a nonprobability sample containing 400 men and 600 women. Suppose external information (e.g., census data) indicates the total population comprises 1000 men and 1000 women. The sample overrepresents women relative to the population. If obesity rates differ by gender, the crude sample estimate will be biased. If the sampling probability is homogenous within each gender, assigning weights of 1000/400 to men and 1000/600 to women restores the correct gender distribution. The weighted obesity rate eliminates bias attributable to gender imbalance, yielding a more representative population estimate.

This logic extends to multiple auxiliary variables. With $\:m$ auxiliary variables, one constructs an $\:m$-way contingency table, defines cells through cross-classifications of these variables, and assigns each observation a weight equal to the ratio of its cell’s population count to sample count. This procedure requires knowledge of the joint distribution of auxiliary variables in the target population–information typically obtained from census data, prior surveys, or administrative records.

Alternatively, poststratification can be implemented by reversing the order of operations. Rather than weighting individuals and then computing the outcome, one first calculates stratum-specific outcome estimates, then aggregates them using population weights. In the obesity example, suppose the observed obesity rates are 30% among men and 10% among women in the sample. Weighting these stratum-specific estimates by the population gender ratio (1000:1000 = 1:1) yields a population estimate of (0.30 + 0.10)/2 = 0.20 or 20%. This is the approach taken by multilevel regression and poststratification (MRP), which fits regression models to estimate stratum-specific outcomes before aggregating [9].

These two approaches are mathematically equivalent. Suppose there are $\:J$ strata indexed by $\:j=1,\:...,\:J$, with sample count $\:{n}_{j}$ and population count $\:{N}_{j}$ in stratum $\:j$. The estimate obtained by first weight the sample and then computing the average is:

$$\:\frac{\sum\limits_{j=1}^{J}\sum\limits_{i=1}^{{n}_{j}}{(Y}_{i,j}\times\:{N}_{j}/{n}_{j})}{\sum\limits_{j=1}^{J}{N}_{j}}$$

where $\:{Y}_{i,j}$ is the outcome of $\:i$-th participant in $\:j$-th stratum. Alternatively, the estimate obtained by first computing cellwise estimate and then calculating the weighted average is:

$$\:\frac{\sum\limits_{j=1}^{J}{N}_{j}\stackrel{-}{{Y}_{j}}}{\sum\limits_{j=1}^{J}{N}_{j}}$$

where $\:\stackrel{-}{{Y}_{j}}=\sum\limits_{i=1}^{{n}_{j}}{Y}_{i,j}/{n}_{j}$ denotes the sample mean in stratum $\:j$. Two formulas give the same results.

Despite this mathematical equivalence, the two implementations differ in practical flexibility. Individual-level weights, once computed, can be applied across multiple analyses examining different outcomes. In contrast, the stratum-aggregation approach generates outcome-specific estimates that are less readily transferable to other analyses.

### Parallels in assumptions

Let $\:X$ denote the auxiliary variables used as poststratifiers and $\:{{\updelta\:}}_{i}\in\:\left\{\mathrm{0,1}\right\}$ indicate whether individual $\:i$ is included in the sample ($\:{{\updelta\:}}_{i}=1$ if included). For simplicity, assume all components of $\:X$ are categorical. For poststratification to yield valid inference about the target population, three assumptions must hold:

1. Conditional Independence. Conditional on $\:X$, sample inclusion is independent of the outcome [10, 11]. Formally, $\:Y\perp\:\delta\:|X$. If sample inclusion is affected by the outcome even after conditional on $\:X$, irreparable bias may appear: Suppose we wish to measure obesity rate. If all obese individuals avoid participation due to their obesity status, the sample obesity rate is zero, and no reweighting scheme can recover the true population rate.
2. Positivity. Every stratum must contain at least one sampled observation [11], expressed as for all *x*,$\:\hspace{0.25em}P\left({\updelta\:}=1|X=x\right)>0$.Without positivity, certain strata have no empirical information, making population-level inference impossible.
3. Consistency. Sample inclusion itself must not affect outcomes. Formally, $\:Y={Y}^{{\updelta\:}=0}={Y}^{{\updelta\:}=1}$, meaning the counterfactual outcome under sampling equals the counterfactual outcome under non-sampling. When estimating obesity rate, if individuals selected for health screening lose weight in anticipation of participation, their observed obesity status differs from what it would have been absent selection. Even a probability sample would then fail to represent the target population, as the act of sampling induces outcome changes. Although we do not go into detail, this assumption aligns with the basic concept of sampling theory: individuals’ outcome value ($\:{Y}_{i}$) is fixed, whereas their sample inclusion ($\:{{\updelta\:}}_{i})$ is random [12]. From this perspective, this assumption is a natural requirement.

Under the three assumptions above, poststratification enables valid population inference. Consider first the approach of weighting individuals. Subjects in stratum $\:j$ receive weight $\:{N}_{j}/{n}_{j}=1/\left({n}_{j}/{N}_{j}\right)$ which equals $\:1/\widehat{P}\left(\delta\:=1|X={x}_{j}\right)$. The weighted estimator then identifies the population mean:

$$E\left[\frac{Y\cdot\:I\left(\delta\:=1\right)}{P\left(\delta\:=1|X\right)}\right]=E\left[E\left[\frac{Y\cdot\:\delta\:}{P\left(\delta\:=1|X\right)}\Bigg|X\right]\right]$$

$$=E\left[E\left[\frac{\delta\:}{P\left({\updelta\:}=1|X\right)}\Bigg|X\right]E\left[Y|X\right]\right]$$

$$=E\left[E\left[Y|X\right]\right]=E\left[Y\right]$$

Alternatively, computing stratum-specific estimates and aggregating with population weights $\:{N}_{J}/N=\widehat{P}\left(X={x}_{j}\right)$ produces:

8 $$\sum\limits_{j}E\left[Y|X={x}_{j},{\updelta\:}=1\right]P\left(X={x}_{j}\right)=\sum\limits_{j}E\left[Y|X={x}_{j}\right]P\left(X={x}_{j}\right)=E\left[Y\right]$$

**Fig. 1:**
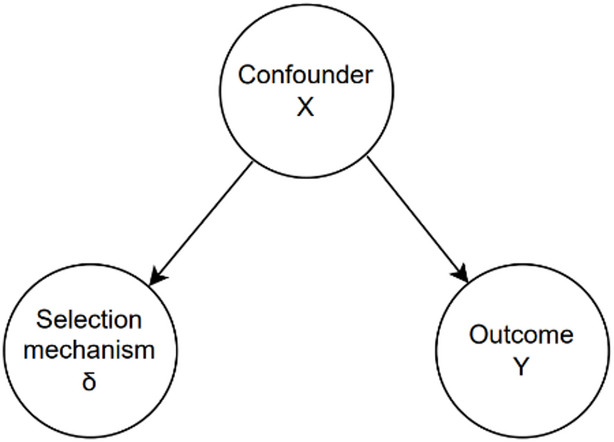
Graphical representation of the conditional independence assumption. Conditional independence holds when the selection mechanism $\:\delta\:$ has no direct pathway to the outcome $\:Y$ and conditioning on $\:X$ blocks all backdoor paths between them

Although causal inference targets counterfactual contrasts and poststratification targets population distribution, the conceptual parallels between poststratification and causal inference become apparent when we consider the sample inclusion indicator $\:{\updelta\:}$ as treatment variable $\:A$. Most notably, the conditional independence assumption corresponds to conditional exchangeability. Under our assumption $\:Y={Y}^{{\updelta\:}}$, the conditional independence $\:Y\perp\:{\updelta\:}|X$ can be interpreted as $\:{Y}^{{\updelta\:}}\perp\:{\updelta\:}|X$ (Fig. 1).

The positivity assumption translates directly between contexts. Just as standard g-methods require at least one subject with $\:A=a$ for each level of $\:X$, poststratification requires that samples exist for each stratum of the auxiliary variables $\:X$. However, poststratification only requires positivity for $\:{\updelta\:}=1$ (sample inclusion), since inference focuses exclusively on representing the target population rather than comparing outcomes between observed and unobserved groups.

The consistency assumption requires a more stringent formulation in the poststratification context. While standard g-methods employ consistency to link observed and counterfactual outcomes across treatment contrasts, poststratification uses $\:{\updelta\:}$ purely as a selection indicator rather than a substantive intervention. This necessitates the stronger assumption $\:Y={Y}^{{\updelta\:}=0}={Y}^{{\updelta\:}=1}$, asserting that sample inclusion itself does not alter outcomes. This requirement represents a conceptual trade-off: the relaxed positivity condition (no need for $\:{\updelta\:}=0$ observations) comes at the cost of a more restrictive consistency assumption that rules out any reactive effects of the sampling process.

### Parallels of implementation methods

The structural parallel between poststratification and causal inference extends naturally to their solution methods. Individual-level weighting followed by outcome estimation is equivalent to IPW, while stratum-specific estimation followed by population-weighted averaging is equivalent to standardization. That these two poststratification methods yield identical results is therefore unsurprising–it is a direct consequence of the well-known equivalence between IPW and standardization under saturated models, when identifiability conditions hold [8].

Consider first the individual-level weighting approach. Subjects with covariate pattern $\:X={x}_{j}$ receive a weight of $\:1/{p}_{j}=1/\left({n}_{j}/{N}_{j}\right)$, where $\:{p}_{j}$ represents the proportion of individuals with $\:X={x}_{j}$ in the target population who were included in the sample. This quantity can be interpreted as $\:{p}_{j}=\widehat{P}\left({\updelta\:}=1|X={x}_{j}\right)$, the estimated probability of sample inclusion conditional on covariates. Viewing $\:{\updelta\:}=1$ as a form of treatment assignment, weighting observations by $\:1/{p}_{j}$ is mathematically identical to IPW in causal inference [13].

A methodological distinction emerges in how these weights are estimated. IPW estimation typically employs model-based approaches, such as logistic regression with treatment $\:A$ as the outcome and covariates $\:X$ as predictors, to estimate propensity scores. This parametric approach is feasible because positivity ensures that both treated ($\:A=1$) and control ($\:A=0$) groups are observable. In contrast, poststratification estimates weights nonparametrically using direct cell counts from the sample and auxiliary information about covariate distributions in the target population. Since the unsampled group ($\:{\updelta\:}=0$) is inherently unobservable, poststratification leverages external knowledge of the target population’s covariate distribution $\:P\left(X={x}_{j}\right)$ to construct weights without modeling $\:P\left({\updelta\:}=1|X\right)$ parametrically. 

Next, consider the approach where stratum-specific estimates are computed first, then aggregated using population-level weights. The mathematical derivation from Eq. (8) mirrors the standardization procedure in Eq. (6) to (7). The key distinction is that poststratification requires external information about the target population’s covariate distribution $\:P\left(X={x}_{j}\right)$, which is unknown from the sample alone, whereas standardization can use the observed covariate distribution.

This approach admits an intuitive interpretation. Suppose $\:X$ encompasses not merely the confounders between $\:Y$ and $\:{\updelta\:}$, but all variables determining the selection mechanism. Under this assumption, all individuals with $\:X={x}_{j}$ share identical sample inclusion probabilities. Consequently, the observed sample within each stratum $\:X={x}_{j}$ constitutes an SRS from that stratum in the target population, with inclusion probability $\:{p}_{j}={n}_{j}/{N}_{j}$. This scenario is equivalent to standard stratified random sampling, which ensures consistent estimation of population parameters.

An important practical consideration concerns which variables to include as auxiliary variables. Variables that affect sample inclusion but not the outcome need not be included in $\:X$ [14]. While omitting such variables means the sample remains unrepresentative with respect to these characteristics, this discrepancy does not bias inference about the outcome of interest. Conversely, including unnecessary variables for poststratification creates numerous small cells, inflating variance and potentially violating positivity when some cells contain no observations [15]. Conditional independence is sufficient. There is no need to achieve perfect representativeness on all dimensions.

This principle parallels variable selection in propensity score estimation for IPW in causal inference. Non-confounding predictors of treatment assignment need not be included when estimating propensity scores. Indeed, Hernán and Robins emphasize that propensity scores need not be estimated with excessive precision, as overfitting the propensity score model by including too many variables can actually degrade performance [8].

### Interpretation between standardization and poststratification

As demonstrated above, standardization and poststratification share nearly identical computational procedures, creating the possibility of interpreting the same calculation through two distinct theoretical frameworks. Suppose we wish to compare obesity rates between individuals with and without depression. Because the two groups differ in their covariate distributions, we weight the data so that covariate distributions match those of a standard population (e.g., all Korean adults in 2023) before making comparisons. This raises a conceptual question: what distinguishes interpreting this procedure as direct standardization in epidemiology from interpreting it as poststratification (specifically, the stratum-specific estimation approach followed by weighted averaging)?

When depression is denoted as $\:A=1$, interpreting this process as direct standardization proceeds as follows. For example, to standardize the obesity rate in the depressed group:

$$E\left[{Y}^{A=1}\right]=\sum\limits_{x}E\left[{Y}^{A=1}|X=x\right]P\left(X=x\right)$$

$$=\sum\limits_{x}E\left[{Y}^{A=1}|X=x,A=1\right]P\left(X=x\right)$$

9 $$=\sum\limits_{x}E\left[Y|X=x,A=1\right]P\left(X=x\right)$$

10 $$=\sum\limits_{x}E\left[Y|X=x,A=1,{\updelta\:}=1\right]P\left(X=x\right)$$

where $\:P\left(X=x\right)$ denotes the covariate distribution in the standard population. Under conditional exchangeability given $\:X$, these steps estimate the counterfactual obesity rate that would occur if the entire standard population were depressed. With the same procedure on $\:A=0$, we can compare standardized obesity rate among depressed and non-depressed populations. However, direct standardization typically proceeds from Eqs. (9) to (10) without explicitly considering the distinction between observed and unobserved patients. This implicitly assumes that conditioning on $\:X$ and $\:A$ is sufficient to achieve conditional independence between and $\:{\updelta\:}$ and $\:Y$.

In contrast, suppose the same procedure is interpreted as poststratification with sample inclusion denoted as $\:{\updelta\:}=1$. This framework considers only depressed subsample ($\:A=1$) from the outset and presupposes that the entire standard population consists of depressed individuals, with only a subset observed in the sample. In this case, $\:Y={Y}^{A=1}$ and

$$E\left[Y\right]=\sum\limits_{x}E\left[Y|X=x\right]P\left(X=x\right)$$

$$=\sum\limits_{x}E[Y\left|X=x,{\updelta\:}=1\right]P\left(X=x\right)$$

under the assumption that $\:X$ comprises predictors of both sample inclusion $\:{\updelta\:}$ and obesity status $\:Y$ (NOT confounders between depression $\:A$ and obesity). These steps estimate the obesity rate for the standard population. Under analogous assumptions, we can similarly obtain a poststratified estimate of non-depressed ($\:A=0$) population.

**Fig. 2:**
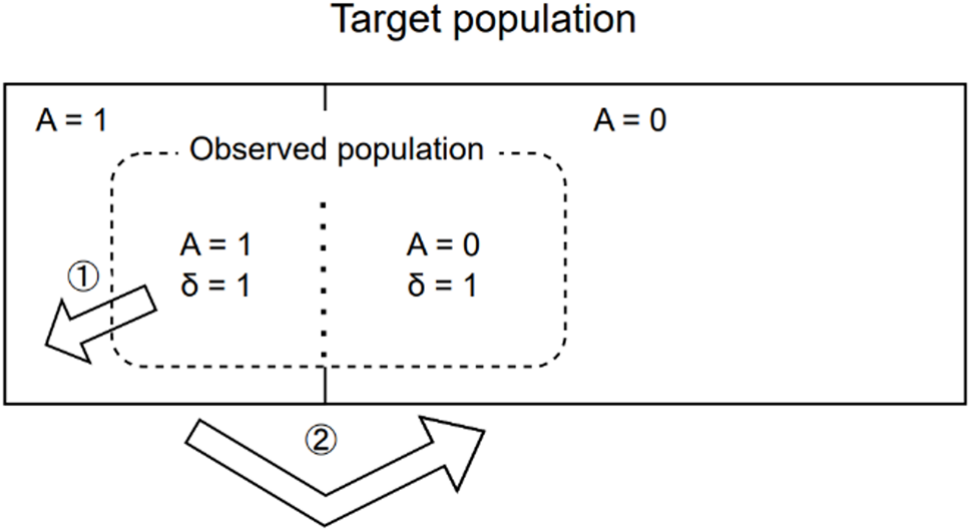
Ideal two-stage procedure for comparing groups from nonprobability samples

Interpretation depends on the context. In reality, unobserved depressed patients would affect the obesity rate (violating the direct standardization assumption) and the standard population contains both depressed and non-depressed individuals (violating the poststratification assumption). Ideally, if auxiliary variable distributions for the complete depressed and non-depressed populations were available, the proper approach would proceed in two stages. First, apply poststratification separately to the observed depressed and non-depressed samples, weighting each to represent their respective complete populations. Second, use direct standardization to compare obesity rates between these poststratification-adjusted groups, ensuring comparability by standardizing to a common covariate distribution (Fig. 2).

First, apply poststratification separately to each exposure group to generalize from the observed sample ($\:A=1,\:\delta\:=1$ and $\:A=0,\:\delta\:=1$ to $\:A=1$ and $\:A=0$). Second, apply direct standardization to ensure comparability between $\:A=1$ and $\:A=0$ groups.

### Empirical illustration: KoGES smoking prevalence

Consider estimating the proportion of current smokers using data from Korean Genome and Epidemiology Study (KoGES) [7]. KoGES is a cohort study conducted by the Korea Disease Control and Prevention Agency’s National Institute of Health since 2001 to identify risk factors for chronic diseases common among Koreans and to provide scientific evidence for personalized and preventive medicine. Participants in this cohort consists of adults aged 40–69 years as of 2001. Because they represent a nonprobability sample of Korean adults aged 40–69, their distribution of auxiliary variables–for example, sex and age–differs from that of the general population obtained from census data (Table 1) [16]. Age and sex are associated with both sample inclusion and smoking behavior [17], making them appropriate auxiliary variables for poststratification adjustment. Therefore, the crude smoking rate among KoGES participants (25.7%) would not represent the smoking rate of all Korean adults aged 40–69 in 2001.

**Table 1:** Distribution of age, sex, and smoking rate of Korean adults aged 40–69 in 2001

Age	Sex	Census^a^	KoGES	Smoking rate^b^
40–49	Men	3,886,462 (25.2%)	2354 (23.5%)	53.1%
40–49	Women	3,758,072 (24.4%)	2357 (23.5%)	3.6%
50–59	Men	2,208,310 (14.3%)	1234 (12.3%)	48.0%
50–59	Women	2,245,444 (14.6%)	1383 (13.8%)	3.1%
60–69	Men	1,493,790 (9.7%)	1170 (11.7%)	44.3%
60–69	Women	1,821,163 (11.8%)	1532 (15.3%)	4.9%
Total		15,413,241	10,030	25.7%

*KoGES* Korean Genome and Epidemiology Study

^a^Census data from Ministry of Public Administration and Security, ^b^Smoking rate among KoGES participants

To apply poststratification, participants in each age-by-sex cell receive a weight equal to the population count divided by the sample count. For example, men aged 40–49 receive a weight of 3,886,462/2354 = 1651.0. The smoking rate of this weighted sample is 26.5%, which is more representative value of the general population with respect to age and sex. For reference, a nationally representative probability sample of the Korean population in 2001 (the Korea National Health and Nutrition Examination Survey [18]) yielded a smoking rate of 29.7% (95% CI: 28.2–31.3) among adults aged 40–69.

## Discussion

Recognizing poststratification through the lens of causal inference provides a principled methodological foundation for making valid population-level inferences from nonprobability samples. By treating sample inclusion as analogous to treatment assignment, the familiar apparatus of causal inference provides immediate insight into when and why poststratification succeeds. In addition, this theoretical bridge reveals that two poststratification strategies correspond to IPW and standardization.

The causal inference framework provides several practical insights that go beyond what poststratification methods alone offer. First, the formal mapping of assumptions clarifies that the key requirement for valid poststratification is conditional independence — not representativeness on all measured variables. This distinction provides principled criteria for selecting auxiliary variables rather than relying on ad hoc choices. Second, this connection enables researchers to interpret poststratification results with more explicit recognition of limitations. Just as no residual confounding remains an ideal in observational study, perfect representation of the target population through poststratification is unattainable, as unobserved factors invariably influence both sample inclusion and outcomes of interest. In particular, both no residual confounding and conditional independence cannot be validated within given data [19, 20]. Consequently, just as epidemiologists exercise appropriate caution when asserting causality, researchers employing poststratification must temper claims of representativeness. The prudent statement is not that a sample “represents the target population” but rather that it has been made representative with respect to specific auxiliary variables [21, 22].

While our illustration prioritized expositional simplicity by assuming discrete covariates with known joint distributions, the framework is adaptable to more complex data structures. When joint distributions are unavailable, methods such as iterative proportional fitting (raking) allow for adjustment based on marginal distributions [23]. Furthermore, continuous auxiliary variables can be incorporated using calibration weighting to align sample moments with those of the target population [24]. Recent advancements in causal inference, such as entropy balancing, mirror these established survey methods by employing similar calibration techniques to handle continuous covariates [25]. This convergence further reinforces the methodological bridge between survey sampling and causal inference.

In our empirical illustration, we selected age and sex as poststratifiers based on their established roles as common determinants of both smoking behavior [17] and study participation. However, adjusting for these demographic factors alone may not fully satisfy the conditional independence assumption. Thus, the poststratified estimate should be interpreted not as a guaranteed recovery of the true population prevalence, but as an estimate improved by removing biases attributable to age and sex. This partial correction is empirically demonstrated in our results, where the poststratified estimate (26.5%) falls between the unadjusted estimate (25.7%) and the benchmark (29.7%). From a causal inference perspective, the remaining discrepancy represents residual sampling bias, analogous to residual confounding. Adopting a causal inference framework encourages such transparency: prompting researchers to use domain knowledge to identify potential unmeasured selection factors (e.g., socioeconomic status) and to recognize that further adjustments or sensitivity analyses may be required.

The target population for poststratification inference is not predetermined but depends on the research objectives. For instance, when obesity prevalence is measured among Korean adults in 2020, the most straightforward target population comprises all Korean adults in that year. However, to take an extreme example, the same data could conceptually be treated as a sample from Korean adults spanning 2015–2025, or alternatively as a sample from Asian adults in 2020. The choice of which population to generalize findings to hinges on the plausibility of the research hypotheses and the tenability of underlying assumptions. This flexibility mirrors a broader principle in observational research: while the study sample itself remains fixed, the target population to which investigators seek to extend their conclusions may vary according to the study’s substantive aims and the strength of arguments for external validity.

Beyond poststratification, substantial methodological work has addressed external validity from nonprobability samples more broadly. Cole et al. proposed the use of inverse probability of selection weights to extend findings from RCTs to the general population [13], an approach that was subsequently extended to incorporate inverse odds of sampling weights [26] and augmented inverse probability weighting (AIPW) [27]. Lesko et al. demonstrated that the conditions required for internal validity in observational studies closely parallel those required for external validity [4]. Future work could draw similar formal parallels between these generalizability methods and survey sampling methods more broadly.

## Conclusions

Epidemiology is the study of how disease is distributed in populations and the factors that influence or determine this distribution [28]. From this perspective, exemplary epidemiological research balances internal validity with external validity, extending findings from study samples to broader populations to generate actionable public health insights. Poststratification offers a principled, computationally straightforward approach to enhancing external validity. By elucidating the conceptual connections between poststratification and the causal inference framework familiar to most epidemiologists, we hope this review encourages wider adoption of these methods and more nuanced interpretation of results from nonprobability samples. Future work could explore how poststratification methods relate to the broader literature on generalizability and transportability of results from randomized trials to larger population or nested cohorts.

## Data Availability

The KoGES data that support this study are available upon request and with the permission of Korea Biobank Network (KBN).

## Abbreviations

RCTs: Randomized controlled trials
ATE: Average treatment effect
SRS: Simple random sample
IPW: Inverse probability weighting
KoGES: Korean Genome and Epidemiology Study
MRP: Multilevel regression and poststratification

## References

[1] Zipf G, Chiappa M, Porter KS, Ostchega Y, Lewis BG, Dostal J. National health and nutrition examination survey: plan and operations, 1999–2010. Vital Health Stat 1. 2013;(56):1–37. 25078429

[2] Valliant R, Dever JA, Kreuter F. Practical tools for designing and weighting survey samples, Second edition edn. Cham, Switzerland: Springer; 2018.

[3] Cornesse C, Blom AG, Dutwin D, Krosnick JA, De Leeuw ED, Legleye S, Pasek J, Pennay D, Phillips B, Sakshaug JW, et al. A Review of Conceptual Approaches and Empirical Evidence on Probability and Nonprobability Sample Survey Research. J Surv Stat Methodol. 2020;8(1):4–36.

[4] Lesko CR, Buchanan AL, Westreich D, Edwards JK, Hudgens MG, Cole SR. Generalizing Study Results: A Potential Outcomes Perspective. Epidemiology. 2017;28(4):553–61. 28346267 10.1097/EDE.0000000000000664 PMC5466356

[5] Lesko CR, Ackerman B, Webster-Clark M, Edwards JK. Target validity: Bringing treatment of external validity in line with internal validity. Curr Epidemiol Rep. 2020;7(3):117–24. 33585162 10.1007/s40471-020-00239-0 PMC7879946

[6] Mercer AW, Kreuter F, Keeter S, Stuart EA. THEORY AND PRACTICE IN NONPROBABILITY SURVEYS: PARALLELS BETWEEN CAUSAL INFERENCE AND SURVEY INFERENCE. Public Opin Q. 2017;81:250–71.

[7] Kim Y, Han BG. Cohort Profile: The Korean Genome and Epidemiology Study (KoGES) Consortium. Int J Epidemiol. 2017;46(2):e20. 27085081 10.1093/ije/dyv316 PMC5837648

[8] Hernan MA, Robins JM. Causal inference: what if, First edition edn. Boca Raton: Taylor and Francis; 2024.

[9] Downes M, Gurrin LC, English DR, Pirkis J, Currier D, Spittal MJ, Carlin JB. Multilevel Regression and Poststratification: A Modeling Approach to Estimating Population Quantities From Highly Selected Survey Samples. Am J Epidemiol. 2018;187(8):1780–90. 29635276 10.1093/aje/kwy070

[10] Smith TMF. Post-Stratification. J Royal Stat Soc Ser D (The Statistician). 1991;40(3):315–23.

[11] Wu C. Statistical inference with non-probability survey samples. Surv Methodol Stat Can. 2022;48(2):283–311.

[12] Fuller WA. Sampling statistics. Hoboken, N.J.: Wiley; 2013.

[13] Cole SR, Stuart EA. Generalizing evidence from randomized clinical trials to target populations: The ACTG 320 trial. Am J Epidemiol. 2010;172(1):107–15. 20547574 10.1093/aje/kwq084 PMC2915476

[14] Pitts AJ, Yomogida M, Aidala A, Gelman A, Chen Q. Multilevel Regression and Poststratification Using Margins of Poststratifiers: Improving Inference for HIV Health Outcomes During the COVID-19 Pandemic. Stat Med. 2025;44(18–19):e70223. 40791168 10.1002/sim.70223 PMC12967158

[15] Brick JM. JM, S.Roth: Identifying Problems With Raking Estimators. In: JSM Proceedings, Survey Research Methods Section: 2003. Alexandria: American Statistical Association; 2003.

[16] Resident Registration Population Status [ https://kosis.kr/statHtml/statHtml.do?orgId=101&tblId=DT_1B04005&conn_path=I2 ].

[17] Kim S, Byun G, Jo G, Park D, Cho SI, Oh H, Kim R, Subramanian SV, Yun S, Oh K, et al. Gender and tobacco epidemic in South Korea: implications from age-period-cohort analysis and the DPSEEA framework. BMJ Open. 2022;12(4):e058903. 35414561 10.1136/bmjopen-2021-058903 PMC9006811

[18] Kweon S, Kim Y, Jang MJ, Kim Y, Kim K, Choi S, Chun C, Khang YH, Oh K. Data resource profile: the Korea National Health and Nutrition Examination Survey (KNHANES). Int J Epidemiol. 2014;43(1):69–77. 24585853 10.1093/ije/dyt228 PMC3937975

[19] Shah R, Peters J. The hardness of conditional independence testing and the generalised covariance measure. Ann Stat. 2020;48(3):1514–1538.

[20] Greenland S, Robins JM, Pearl J. Confounding and Collapsibility in Causal Inference. Stat Sci. 1999;14(1):29–46.

[21] Schouten B, Cobben F, Bethlehem J. Indicators for the representativeness of survey response. Surv Methodol. 2009;35(1):101–113.

[22] Ochsner M. Representativeness of Surveys and its Analysis. FORS Guide No. 15, Version 1.0. Lausanne: Swiss Centre of Expertise in the Social Sciences (FORS); 2021. 10.24449/FG-2021-00015.

[23] Deville J-C, Särndal C-E, Sautory O. Generalized Raking Procedures in Survey Sampling. J Am Stat Assoc. 1993;88(423):1013–20.

[24] Deville J-C, Särndal C-E. Calibration Estimators in Survey Sampling. J Am Stat Assoc. 1992;87(418):376–82.

[25] Hainmueller J. Entropy Balancing for Causal Effects: A Multivariate Reweighting Method to Produce Balanced Samples in Observational Studies. Political Anal. 2012;20(1):25–46.

[26] Westreich D, Edwards JK, Lesko CR, Stuart E, Cole SR. Transportability of Trial Results Using Inverse Odds of Sampling Weights. Am J Epidemiol. 2017;186(8):1010–4. 28535275 10.1093/aje/kwx164 PMC5860052

[27] Dahabreh IJ, Robertson SE, Tchetgen EJ, Stuart EA, Hernán MA. Generalizing causal inferences from individuals in randomized trials to all trial-eligible individuals. Biometrics. 2019;75(2):685–94. 30488513 10.1111/biom.13009 PMC10938232

[28] Celentano DD, Szklo M, Farag YMK, Gordis L. Gordis epidemiology, Seventh edition edn. Philadelphia, PA: Elsevier; 2025.

